# Sudden bio-mathematical self-similarity and the uniqueness of human mass societies: from T-patterns and T-strings to T-societies

**DOI:** 10.3389/fpsyg.2023.1157315

**Published:** 2023-05-18

**Authors:** Magnus S. Magnusson

**Affiliations:** Human Behavior Laboratory, School of Health Sciences, University of Iceland, Reykjavik, Iceland

**Keywords:** interaction, T-pattern, mass societies, RNA to DNA-worlds, self-similarity, interactive emergence society, T-society, human uniqueness

## Abstract

With the explosive growth of human knowledge especially in the twenteeth century with even greater facilitation of access to knowledge, the world of even relatively recent great thinkers becomes daunting as seen from a modern viewpoint. Recently, humans ignored the existence of the complex intracellular world of cell organs, giant information molecules such as DNA, societies of specialized worker molecules (proteins), and generally the surprising nanoscale world visible to humanity since only a few decades ago. Moreover, computational power and video technology were inaccessible to all scientists from, for example, Aristotle to Freud, so new views and ideas seem to be expected about phenomena at all scales including nano and human. Some have arrived very recently. Thus urgently needed knowledge about the biology of animal and human behavior received the first Nobel Prize as late as 1973, in Physiology and Medicine, shared by Karl von Frisch, Konrad Lorenz, and Niko Tinbergen. Lorenz's Nobel lecture was entitled “Analogy as a Source of Knowledge” which did not mention self-analogy (self-similarity) as none of the species studied were part of others and knowledge of the nanoscale phenomena at the heart of this article had barely become available. The views and empirical findings presented in this article depend on such recent intracellular nanoscale insights and the development of a set of mathematical patterns, called T-system, of which only two are considered, the self-similar (i.e., parts having a structure similar to the whole) T-pattern and the derived T-string, a T-patterned material string (here, polymer or text). Specially developed algorithms implemented in the THEME^TM^ software for T-pattern detection and analysis (TPA) allowed the detection of interaction T-patterns in humans, animals, and brain neuronal networks, showing self-similarity between animal interaction patterns and neuronal interaction patterns in their brains. TPA of DNA and text also showed unique self-similarity between modern human literate mass societies and the protein societies of their body cells, both with Giant Extra-Individual Purely Informational T-strings (GEIPIT; genomes or textomes) defining the behavioral potentials of their specialized citizens. This kind of society is here called T-society and only exists in humans and proteins, while the self-similarity between them only exists in human T-societies.

## 1. Introduction

This article is a part of the Frontiers Research Topic collection: **“**Behavior and Self-Similarity between Nano and Human Scales: From T-pattern and T-string Analysis (TPA) with THEME to T-Societies” with a common reference free online article “T-patterns, external memory, and mass-societies in proteins and humans: In an eye-blink, the naked ape became a string-controlled citizen” (Magnusson, 2020b). The research presented here has, since the 1970s, created a still-growing set of temporal and spatial (string) mathematical patterns, called the *T-system* as a tool and language for the detection, analysis, and description of behavioral and other biological structures. Here, the focus is on its latest addition, called the T-society, which only implicates two of its earlier patterns, the T-pattern and the T-patterned material string called a T-string (see below).

Discoveries in the biology of animal and human behavior received the first Nobel Prize as late as 1973, in Physiology and Medicine, shared by Karl von Frisch, Konrad Lorenz, and Niko Tinbergen. In his Nobel Prize lecture “Analogy as a Source of Knowledge,” Konrad Z. Lorenz wrote “Ethologists are often accused of drawing *false* analogies between animal and human behavior. However, no such thing as a *false* analogy exists: an analogy can be more or less detailed and hence more or less informative” (Lorenz, 1973, p. 98, or see Lorenz, 1974). “There is, in my opinion, only one possibility of an error that might conceivably be described as the ‘drawing of a false analogy' and that is mistaking a *homology* for an analogy. Homology can be defined as any resemblance between two species that can be explained by their common descent from an ancestor possessing the character in which they are similar to each other” (Lorenz, 1973, p. 99, or see Lorenz, 1974).

Lorenz did not mention self-analogy (self-similarity) as none of the species studied was a part of another. Knowledge was also still limited about intracellular nanoscale phenomena such as the ribosome discovered in 1952 by G. E. Palade who received the Nobel Prize in 1974 for its further study^1^ The central role of the ribosome has since been widely recognized: “The ribosome translates the DNA code into life. The Nobel Prize in Chemistry for 2009 awards studies of one of life's core processes: the ribosome's translation of DNA information into life. Ribosomes produce proteins, which in turn control the chemistry in all living organisms.” ^2^ “At the beginning of the twentieth century, the chemical foundations of life were mysterious. Today we know how many of the most important processes function, all the way down to the atomic level.” The Nobel Prize in Chemistry for 2009 was awarded jointly to V. Ramakrishnan, T. A. Steitz, and A. E. Yonath (Ramakrishnan, 2018) “*for studies of the structure and function of the ribosome*.” ^3^

The ribosome and the RNA World hypothesis^4^ are at the heart of this article.

The empirical and theoretical approach began with a study of both individual and social behavior of other animals from ethological, behavioristic, and linguistic viewpoints with varying emphasis on direct observation in natural settings or on laboratory experiments regarding verbal and non-verbal probabilistic interaction contingencies, as well as the (temporal) syntactic structure of verbal behavior (vocal and written) and creativity. The present study is the latest in a series with mainly an ethological viewpoint (Magnusson, 1975, 1983, 1988, 1989, 1996, 2000, 2004, 2005, 2006, 2009, 2016b, 2017, 2018, 2020a,b, 2022; Magnusson and Beaudichon, 1997; Magnusson et al., 2016). This study began in the 1970s with a study of communication and social organization in social insects and primates (including humans) (Magnusson, 1975) searching for a valid and useful definition of what distinguishes humans from other animals. One suggested answer is presented here, found through the intracellular polymer nanoworld of DNA, RNA, proteins, and ribosomes combined with the T-system concepts of T-string and the latest addition T-society which is the focus of this study.

### 1.1. The mathematical and computational approach

Computers made it possible to search for hidden patterns given adequate mathematical definitions and detection algorithms implemented in computer programs both still in short supply. After considering and testing standard multivariate statistical methods and software (see, for example, Colgan, 1978) available in major statistical software packages, notably, SAS and SPSS (Magnusson, 1983), it appeared that for the analysis of real-time behavioral records, more adequate pattern models were needed with corresponding detection algorithms and software which led to the so-called temporal configuration and later T-pattern (Magnusson, 1983, 1988, 1996, 2000). The detection algorithms were initially developed and implemented as a 3k-line THEME PDP 8 Fortran IV software (Magnusson, 1983), but now the >300k-line versions have been extensively used mostly for temporal T-pattern detection and analysis (TPA) of human, animal, and neuronal brain network interactions (Anolli et al., 2005; Casarrubea et al., 2015, 2022; Nicol et al., 2015; Magnusson et al., 2016; Anguera et al., 2023). This has gradually led to the T-system, a set of mathematical patterns and a language and tool for the analysis and comparison of temporal and spatial structure across species, scales, and organizational levels (Magnusson, 2020b).

T-pattern detection and analysis (TPA) with the THEME^TM^ software (patternvision.com) has been applied in many areas with studies published in two dedicated edited volumes (Anolli et al., 2005; Magnusson et al., 2016) and in many studies as already reviewed (Casarrubea et al., 2015; Consuelo et al., 2022; Anguera et al., 2023, and see related, Casarrubea et al., 2018, 2022).

### 1.2. The T-pattern model and detection algorithm

The following sections describe essential aspects of the T-pattern model and detection algorithms while referring to recent free open-access online publications (for example, Magnusson, 1996, 2000, 2017, 2020a,b).

### 1.3. T-data

**Figure 1:**
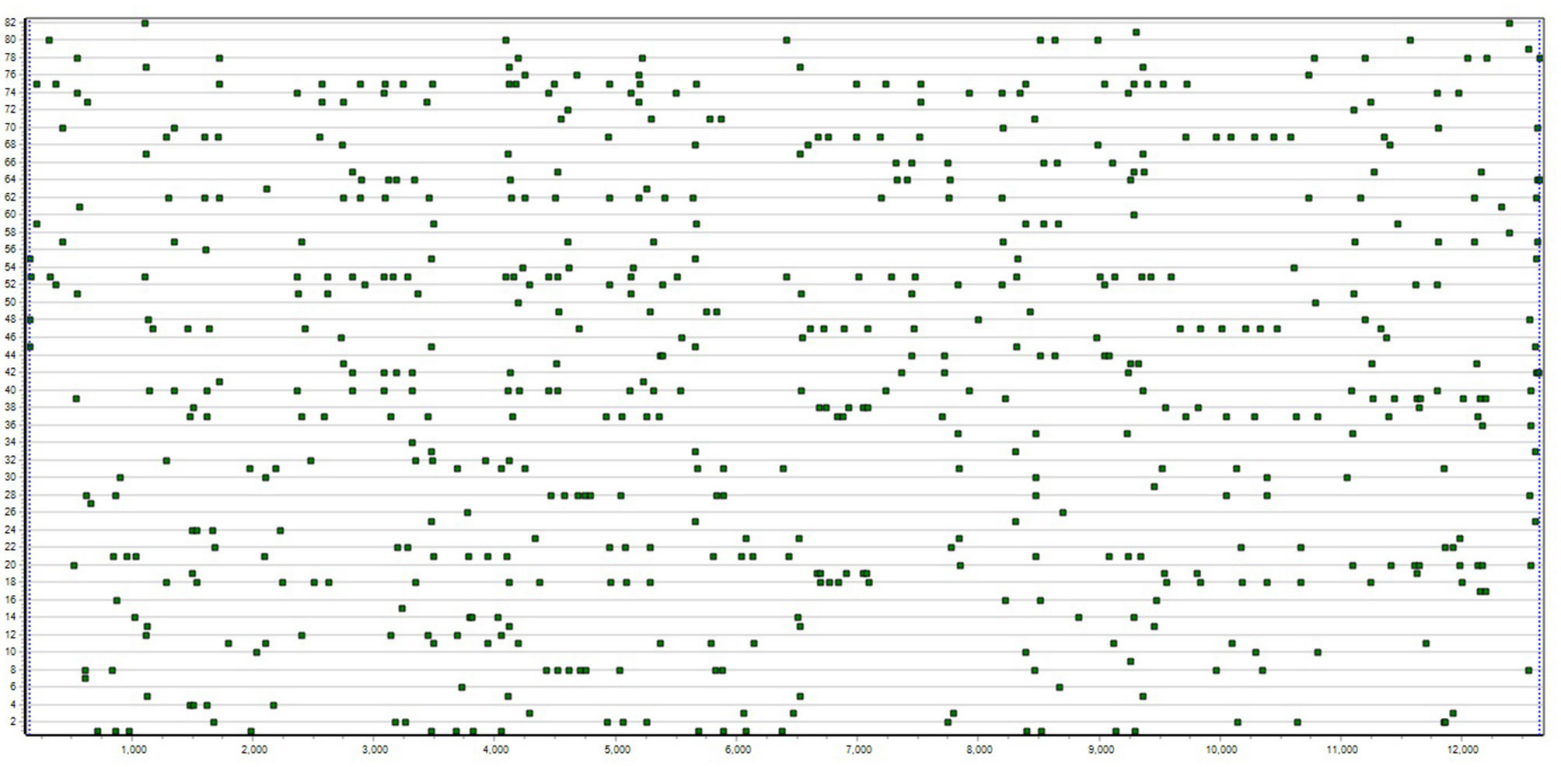
T-data example shows 82 occurrence series each for the different behavior of one of two children exchanging and playing with a toy for 13 and a half minutes [see for example, Magnusson (2020a)].

The type of data, called T-data, referred to by all T-system definitions is labeled series of positions on a single discrete dimension, 1 to T, here, in time or on strings. The series label specifies the type of event in time or element in a string (see Figure 1).

### 1.4. The T-pattern

The T-pattern, which is a self-similar binary tree, that is, has the same statistical relationship at each non-terminal node. It attempts to consider several structural aspects of behavior such as order (including concurrence), modularity, and hierarchy.

A T-pattern, Q, is defined as m ordered components, X_1..m_, recurring on a single discrete dimension within [1, T], where each component is either a T-data series or a T-pattern, noted as follows:


$$\begin{array}{l} Q:X_{1}\approx dt_{1}X_{2}\approx dt_{2}...X_{i}\approx dt_{i}X_{i+1}\ldots X_{\text{m}-1}\approx dt_{\text{m}-1}X_{\text{m}} \end{array}$$

(m = length), where the **≈dt** (0 ≤ dt) terms stand for the approximate distances between the consecutive components X of the pattern when it occurs within T-data, each significantly invariant relative to a zero hypothesis of independent random distribution of each component with constant baseline probability per unit time given by its number of occurrences divided by T.

In terms of the intervals of variation of the **≈dt** terms, the definition comes closer to the currently used detection algorithms:


$$\begin{array}{l} Q:X_{1}{\left[d_{1},d_{2}\right]}_{1}X_{2}{\left[d_{1},d_{2}\right]}_{2}...X_{i}{\left[d_{1},d_{2}\right]}_{i}X_{i+1}\ldots X_{\text{m}-1}{\left[d_{1},d_{2}\right]}_{\text{m}-1}X_{\text{m}} \end{array}$$

(m = length), where the intervals [ d_1_, d_2_ ]_*i*_; 0 ≤ d_1_ ≤ d_2_ are the intervals within which ≈dt_i_ varies.

### 1.5. T-pattern detection and analysis (TPA)

This section describes the essentials of the T-pattern detection algorithm implemented in the Theme software.

The current binary-tree bottom-up search algorithm relies on finding in T-data one or more pairs of series related by so-called critical intervals and then adding their occurrence series to T-data, thus including them in the continued search for more pairs and possible pairs of pairs, etc.

An evolution algorithm gradually builds the patterns while removing redundancy. The binary tree approach has allowed the detection of numerous and complex T-patterns, but as shown elsewhere, this does not guarantee the detection of all T-patterns in the data requiring possibly trinary or higher trees (Magnusson, 2020a).

All the X terms, and especially in small data, can thus be simple events and the T-pattern may have no inherent modular, hierarchical, or self-similar structure as suggested by pattern diagrams, which may simply be artifacts of the binary tree detection procedure, but this can normally be seen in T-pattern diagrams with non-terminal nodes (sub-patterns) not being more frequent than the whole pattern.

Self-similarity, meaning that a part has the same structure as the whole (see, for example, dictionary.com), is a central aspect of fractal structures which in mathematics are infinite, but in nature, they do not have infinite levels “self-similarity over a range of scales is a hallmark of fractal geometry,” (Kautz, 2011, p. 280) and “Physical fractals typically display statistical self-similarity over scales differing by just a few factors of 10. Nonetheless, as Mandelbrot first observed, it is extremely useful to recognize the fractal properties of natural shapes” (Kautz, 2011, p. 293).

In his book “The Fractal Geometry of Nature,” Mandelbrot implicates self-similarity in many ways, for example:

“Furthermore, most fractals in this Essay are invariant under certain transformations of scale. They are called scaling. A fractal invariant under ordinary geometric similarity is called self-similar” (Mandelbrot, 1983, p. 18).

**Figure 2:**
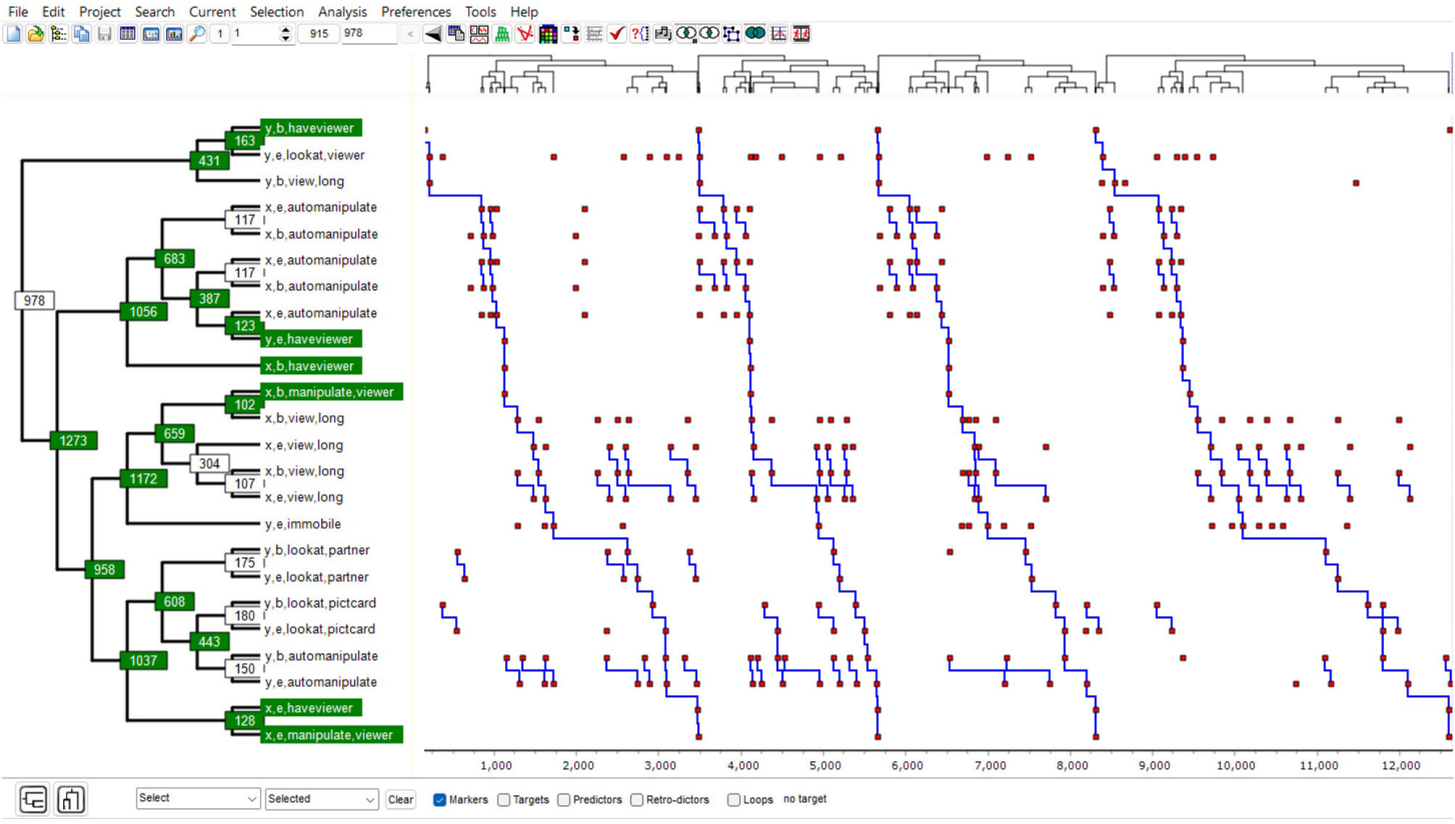
The t-pattern diagram example shows a T-pattern detected with THEME 6 in the data shown in Figure 1 [see, for example, Magnusson (2017)].

The T-pattern is a self-similar tree structure, that is, with the same critical interval relationship (CIR) at each non-terminal node; moreover, it recurs with significant translation symmetry (as defined also by the critical interval relationship, CIR). It can thus also be seen as a particular kind of recurring semi-rigid CIR-based statistical fractal pattern (see Figure 2 and Magnusson, 2017, 2020a,b).

### 1.6. The T-string

The T-string is a material string of length T of elements 1..T, where some of the elements form T-patterns, for example, RNA, DNA, proteins, and text (see Magnusson, 2020b, section 5). The T-string is thus seen as a material version of a discrete-time scale (see T-data above) of the same length and containing T-patterns.

## 2. The RNA world hypothesis

**Figure 3:**
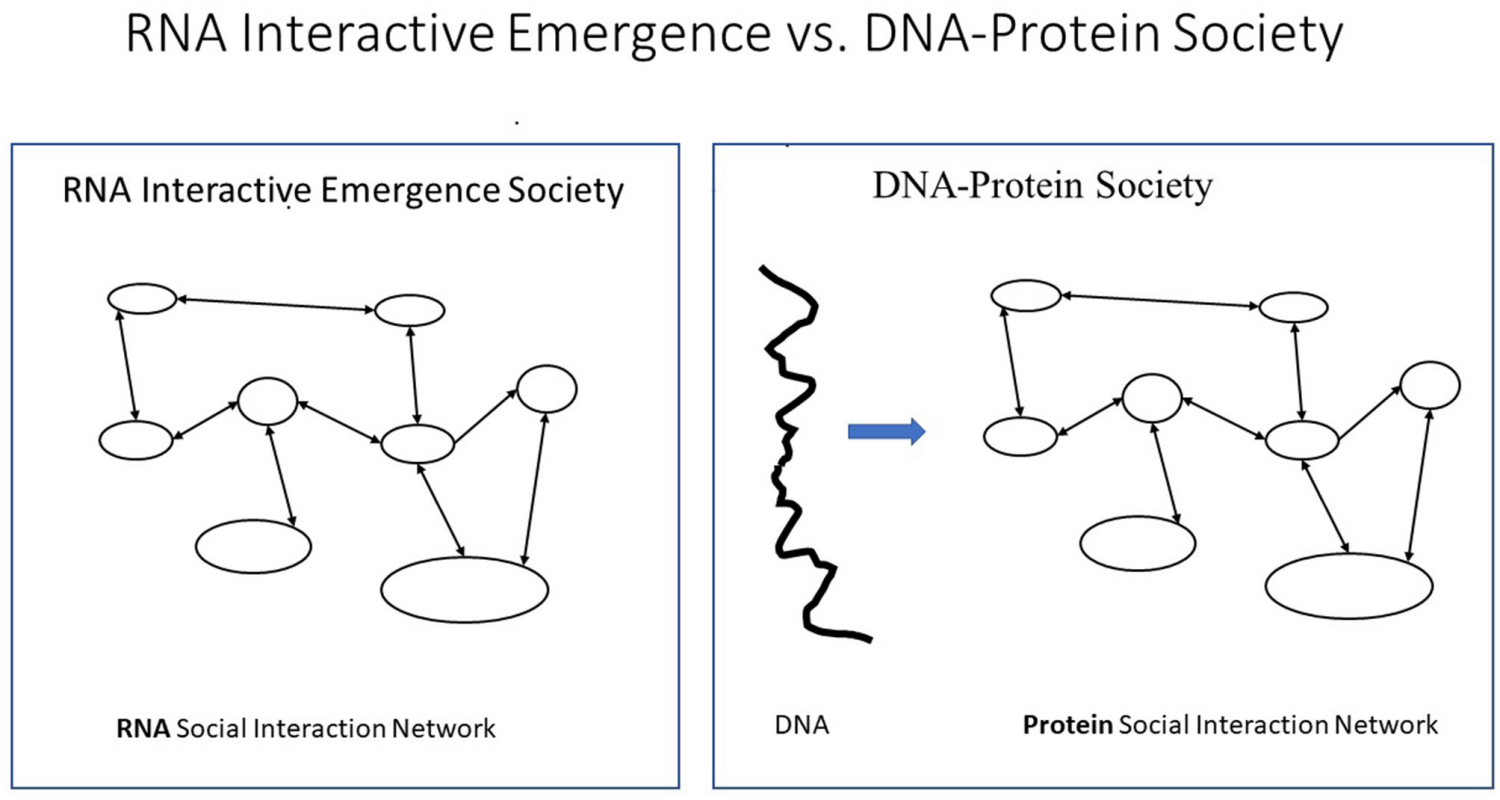
Interactive Emergence Society (IES) vs. T-society. These two figures, left and right, represent two types of societies. **(Left)** An Interactive Emergence Society where citizens interact, and a social structure emerges. **(Right)** An external string partly determines the characteristics of the interacting individuals. The first **(left)** describes the RNA world and animal (including illiterate humans) societies. The other **(right)** called a T-society is only found in the cells of the DNA world and in human modern mass societies.

According to the RNA World hypothesis (see, for example, Pressman et al., 2015), billions of years ago, a major evolutionary transition occurred from one polymer T-string world to another, from the RNA world, where RNA polymers took care of both information storage and work, to a DNA world, where inert DNA polymers only store information, while proteins do most of the work. Thus, DNA became an inert giant extra-individual purely informational storage polymer (string) outside the proteins (citizens) but defining their structure, behavioral potentials, tendencies, and roles.^5^ A social organization that emerges from interactions between citizens without such giant purely informational extra-individual strings is here called *Interactive Emergence Society (IES)* and is the only kind in the RNA world and in animals and preliterate human beings, see Figure 3.

## 3. The T-society

With the human invention of inert extra-individual purely informational memory, i.e., text, analogous separation happened between work and information determining numerous neuronal brain patterns in the citizens and, thus, much of their behavioral potentials, tendencies, and roles.

The detection of T-strings in DNA and proteins and in the letter strings of texts (Magnusson, 2020a,b) shows similarities in patterning across very different scales and levels of biological organization. The similarity between protein and literate human mass societies was thus recognized as each is based on what here is called *Giant Extra-Individual Purely Informational T-string* or *GEIPIT*, determining the characteristics of different types of individuals (members, citizens). The genome in the protein society and the textome (i.e., all text) in a literate human society are, thus, GEIPITs and here together called T-stringomes.

**Figure 4:**
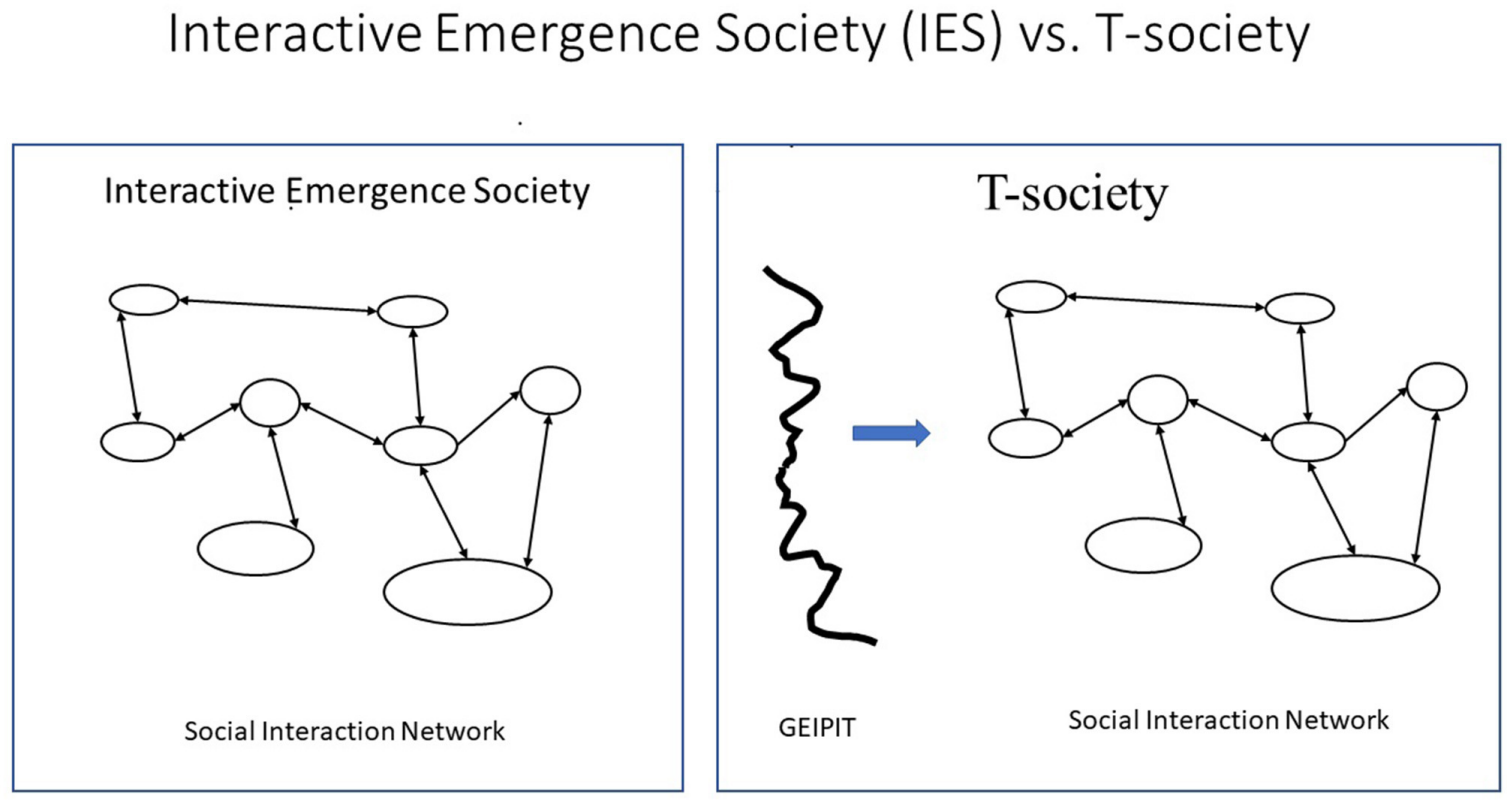
The similarity between protein and literate human mass societies, here called T-societies, was recognized as each is based on what here is called Giant Extra-Individual Purely Informational T-string or GEIPIT, determining the characteristics of different types of individuals (members, citizens). A social organization that emerges from interactions between citizens without GEIPIT is here called Interactive Emergence Society (IES).

This has allowed the definition of the T-system concept *T-society*, as a T-stringome-based society, found only in proteins and literate humans but neither in non-human animals nor illiterate humans (see Wilson, 1975; Hölldobler and Wilson, 2008) (see Figure 4).

### 3.1. Social organization of two kinds and a human paradigm shift

**Figure 5:**
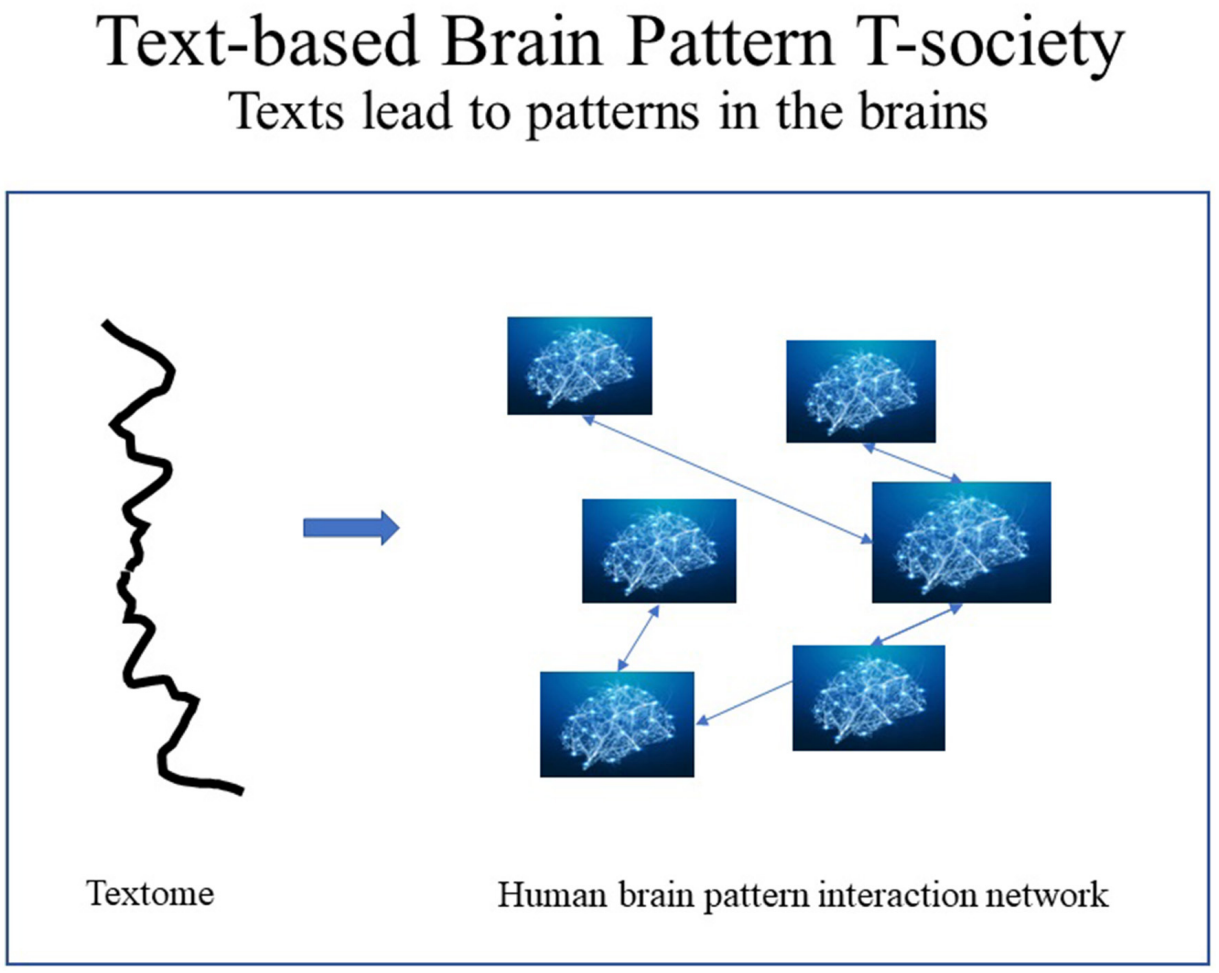
Texts form patterns in the brain for the specialization of citizens.

In human T-societies, text segments, such as curricula, are to brain patterns what genes are to proteins, assuming that the behavior of literate humans is partly determined by brain patterns shaped by exposure to text. Thus, in protein T-societies: DNA → protein 3-D structure → protein behavior, while in human T-societies: text → brain patterns → human behavior. It has been suggested that T-patterns are involved: “because spike-timing is of such theoretical importance, analysis of temporal sequencing in multiple neuronal activity, using such techniques as T-pattern analysis, may be of great value in understanding the mechanisms whereby neuronal networks encode sensory information” (Nicol et al., 2005, p. 74) (see Figure 5).

**Figure 6:**
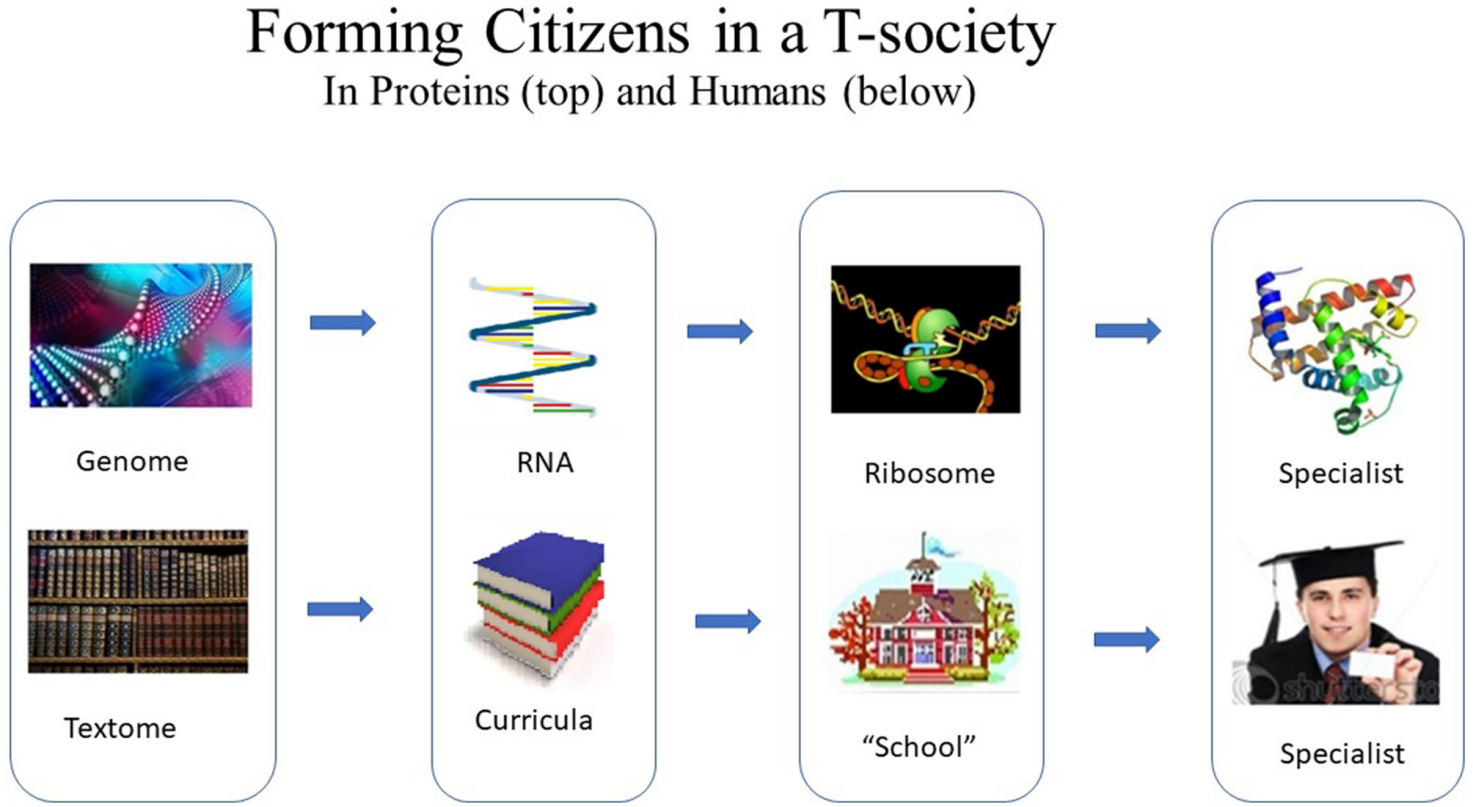
Forming of citizens in T-societies. Process (top) leading from the genome to a specialized protein citizen in an intracellular protein T-society and (bottom) the analogous process from the textome (i.e., all the text) of a human T-society *via* curricula and a “school” (educations system) to a specialized human citizen.

Among the best-known segments for the forming of specialized citizens are the genes of the genome and the curricula used in educational institutions (Magnusson, 2020b). Most of the genome and the textome do not have a citizen-defining function, for example, “non-coding DNA.”^6^ (Figure 6).

The separation of information and action was, thus, essential when the RNA world invented the DNA world and preliterate humans invented the textual world.

Therefore, after billions of years of evolution, this unique self-similarity appeared, in a biological eyeblink but with considerable consequences, really leaving all living creatures behind. Humans, thus, became the **first and only species** to develop the same social organization *paradigm found in every biological cell*, including all those constituting all its citizens. Texts as precise unlimited extra-individual T-string memory suddenly allowed an explosive increase in the specialization of individuals and in human knowledge, science, technology, and law. This revolutionary increase in human abilities, and knowledge of nature and the universe, seems to belong among the very rare major evolutionary transitions (see, for example, Kun, 2021).

**Figure 7:**
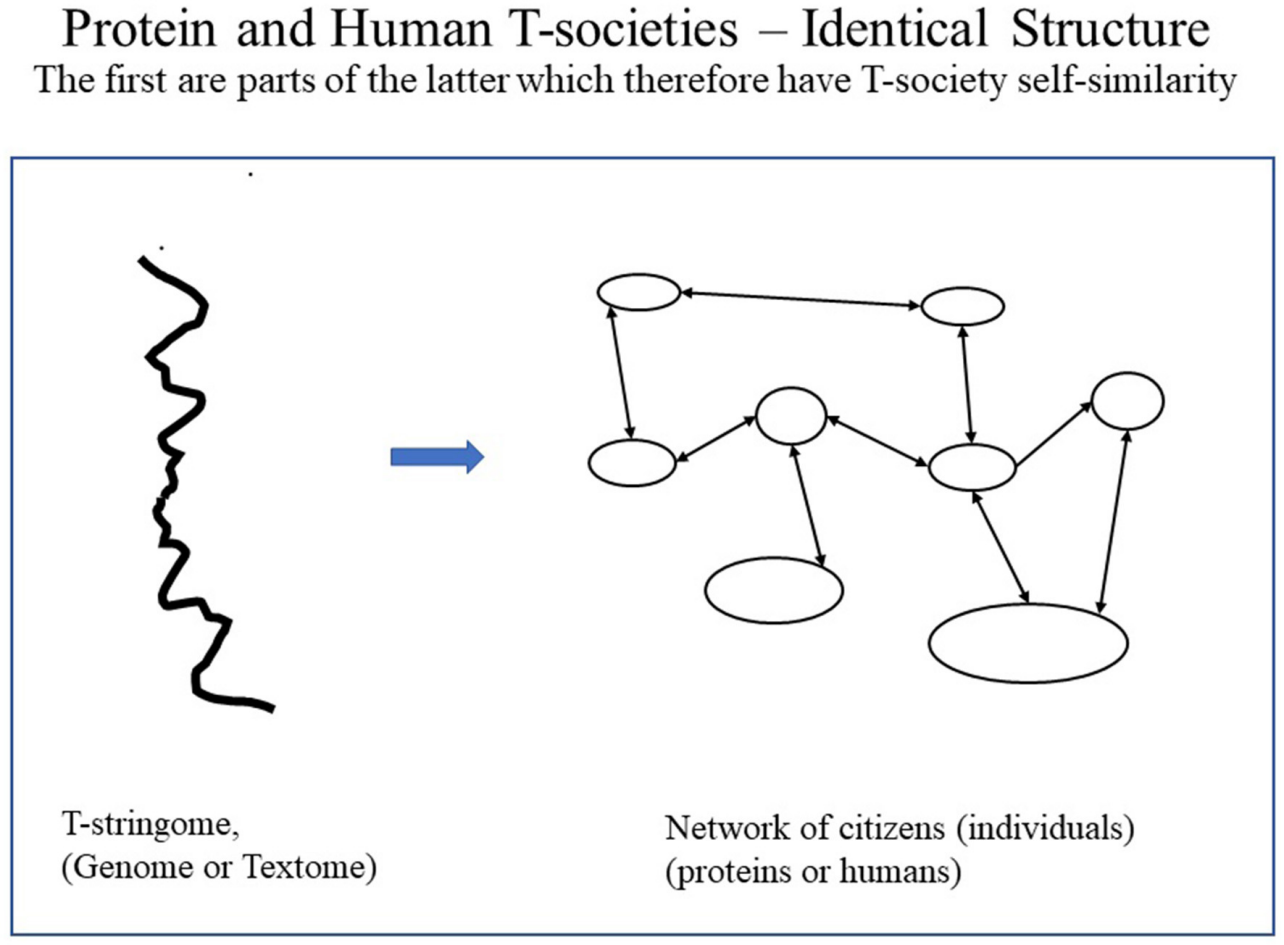
Analogous structure of protein and human T-societies underlining the fact that the first is a part of the other so there is self-similarity.

**Figure 8:**
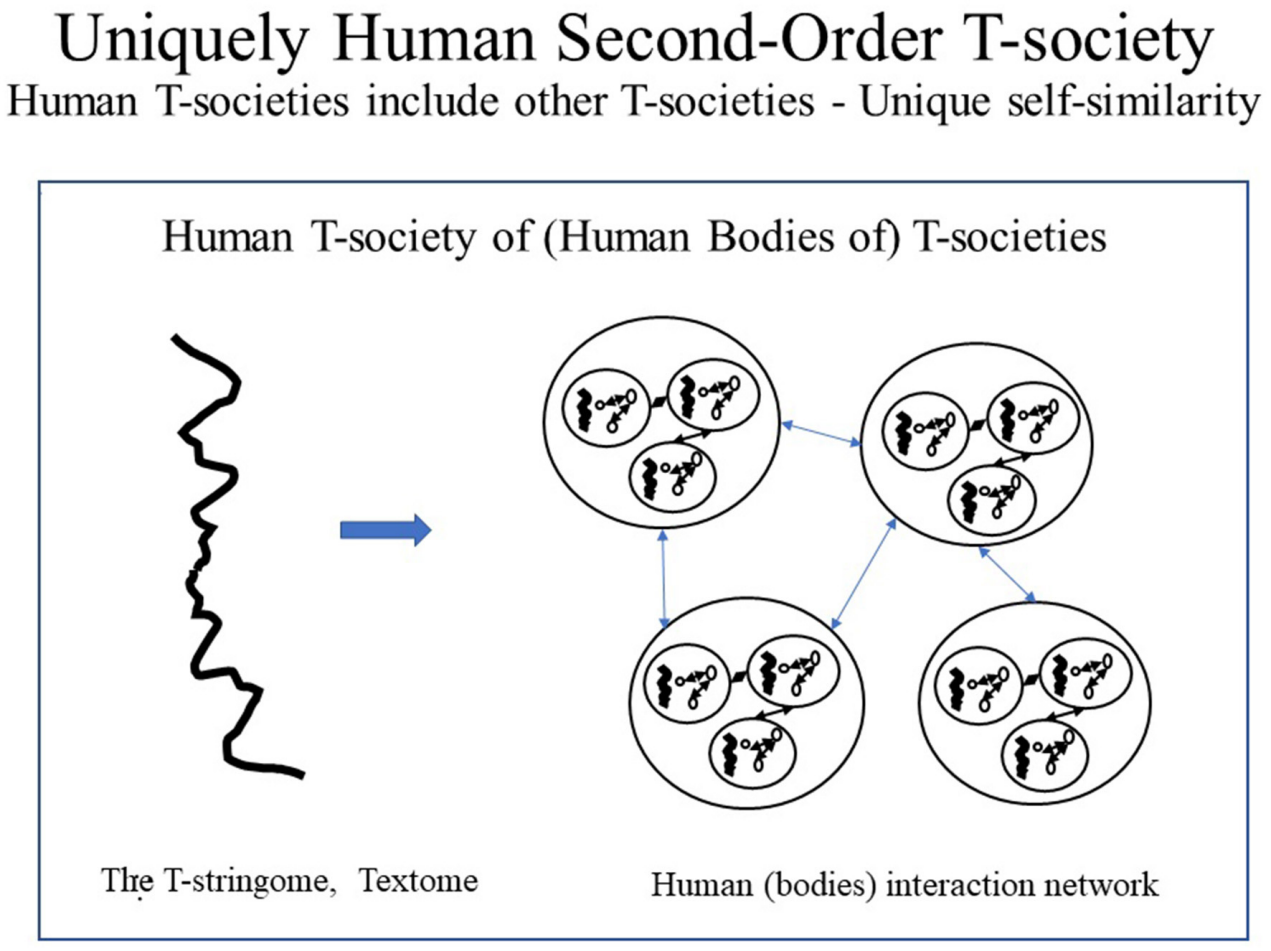
Uniquely Human Second-Order T-society. Human T-societies which are only found in literate humans are unique in having all citizens made of other T-societies, i.e., proteins T-societies. The structure of a human T-society as a whole is the same as the structure of its parts. This self-similarity does not exist in other animals or illiterate humans and is, thus, uniquely human.

**Figure 9:**
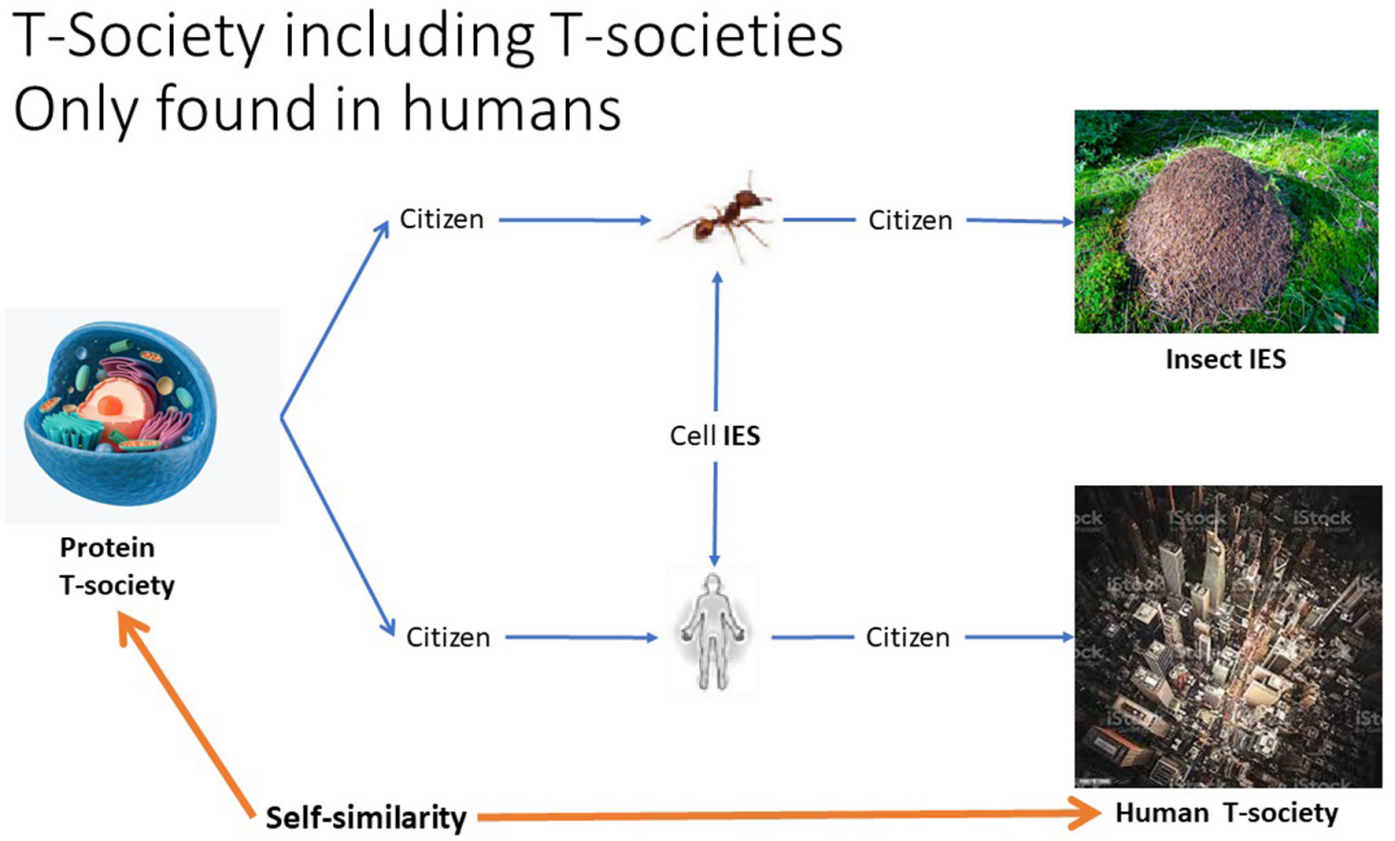
Relationships between Interactive Emergence Societies (IES) exemplified by two different kinds of IES of cells, that is, the bodies of an ant and a human. They make up two different kinds of mass societies, an ant hive (IES) and a human T-society. While a cell (protein T-society) on the left is a component in all the others, it has a self-similarity only with the human T-society, humans being the only species to have created such societies.

As human citizens are composed of protein T-societies (i.e., cells), human T-societies are T-societies of T-societies, here thus called *second-order T-societies*. Such T-society self-similarity is not found in proteins and is, thus, unique to humans (see Figures 7–9). Figure 7 shows protein and human T-societies, analogous structure.

### 3.2. T-societies and the invention of writing

While T-societies have existed in cells for billions of years, the invention of writing, only a few thousand years ago, was a necessary precondition for the development of human T-societies. Simple messaging and bookkeeping were insufficient as standardized texts, and their effective copying and distribution to form the many kinds of specialized individuals (citizens) were needed in complex societies. It would take a very long time to reach the level of protein T-societies as for most of the time since the beginning of writing it has been very different.

“Literacy rates over most of human existence were insignificant. Estimates from the Middle Ages, which are primarily based on the proportion of people who could sign their name on various documents, point to rates below 10 per cent in countries such as China, France, Germany, Belgium and the Netherlands, and even lower levels elsewhere in Europe and across the globe” (Galor, 2022, pp. 63–64).

Only very recently, the now largely text-based educational system began to create countless different kinds of specialists:

“education in pre-industrial Europe was still not geared toward the provision of skills to a mass workforce” (Galor, 2022, p. 65).

In the Industrial Revolution era, this finally changed:

“In England…In 1841, for instance, only 5 per cent of male workers and only 2 per cent of female workers were employed in occupations in which literacy was required. Workers developed skills primarily through on-the-job training,” (Galor, 2022, p. 67).

“In the earliest phase of the Industrial Revolution, literacy and numeracy played a limited role in the production process, and thus the enhancement of these aspects of human capital would have had a limited effect on workers' productivity. Although some workers, supervisory and office personnel in particular, were required to be able to read and perform elementary arithmetical operations, a large portion of the tasks in industry was successfully performed by people who were illiterate. During the subsequent phases of the Industrial Revolution, the demand for skilled labor in the growing industrial sector markedly increased. From here on, and for the first time in history, human capital formation—factors that influence worker productivity, such as education, training, skills, and health—was designed and undertaken with the primary purpose of satisfying the increasing requirements of industrialization for literacy and numeracy as well as mechanical skills among the workforce” (Galor, 2022, p. 67).

### 3.3. Slow spread of literacy

“The global literacy rate currently stands at 87%, up from 12% in 1820. Most developed countries have achieved a 99% literacy rate” World Economic Forum.^7^

If the human invention of extra-individual T-string memory, i.e., text, was important, why did not it spread faster among all *Homo sapiens*? It seems this great tool was like so much else kept by and for ruling subgroups and was among others used to ascertain that order with T-strings sometimes set in stone:

“Hammurabi's Code, for example, established a pecking order of superiors, commoners, and slaves. Superiors got all the good things in life. Commoners got what was left. Slaves got a beating if they complained” Harari (2014, p. 168).

“For most of human history, formal education was available only to a small, privileged section of society” (Galor, 2022, p. 63).

## 4. Discussion

After billions of years of biological evolution, a shift in human social organization gradually became analogous with the protein T-societies in every biological cell, a self-similarity unique not only in the animal world but in all life. A transition that made possible the progress in science and technology that allowed its own discovery.

Human T-societies became gradually possible with the advent of writing (~3500 BC) followed by the creation of texts and gradually more effective means of copying and distribution (recently, printing, and Internet) and, eventually, of standardized curricula used by schools to form the multitude of different brain patterns, and thus behavioral potentials, of the many specialized human citizens.

The mass societies of modern *Homo sapiens* thus seem to have adopted a new social organization paradigm analogous with every cell in their bodies, while different from all other animals and earlier humans even of the same species. This seems to justify rethinking human mass society issues, but, apparently, this has not happened generally judging, for example, by the recent writings of highly esteemed and widely read authors as indicated below by a few glimpses into their writings.

### 4.1. Population sizes, citizen types, and social coherence

Tens of thousands of years after the advent of language, extra-individual T-string memory, i.e., text first appeared in humans. This unique evolutionary transition happening in an eyeblink has allowed a great increase in the number of different types of citizens and population sizes, with ever-faster growth of human mass-social potential and possibly even explaining the recent shrinking of the human brain (DeSilva et al., 2021). Billions of citizens of thousands of different types in some protein societies far exceed any animal society other than modern human mass societies, such as in China and India, each exceeding a billion individuals and with new texts and kinds of specialists constantly being created (Milo and Phillips, 2015, p.105). Moreover, manyfold the size of the largest non-human mass societies, those of social insects that are interactive emergence societies (IES), not T-societies (Hölldobler and Wilson, 2008), same as with animal bodies seen as societies of cells, which are IES, not T-societies.

“The world population tripled between 1500 and 1900, to an estimated 1564 million” (see https://worldmapper.org/maps/population-year-1900/). With the population of China and India, each now reaching nearly that size, how could such mass-social cohesion and collaboration be possible given the evolutionary history of the human species?

Among those who recently have posed this question and suggested answers are Dunbar (2022) and Harari (2014), but neither mentions the kind of nanoscale phenomena or self-similarity described above. While, of course, recognizing the considerable importance of writing, both, and especially Dunbar, treat human mass societies as primate interactive emergence societies (IES).

### 4.2. Robin Dunbar, brains, religion, and population size

After decades of study of relations between brain structure, social interaction, social organization, and population size in primates, both non-human and human, Robin Dunbar wrote the following part:

“The core of the social brain hypothesis is a simple linear relationship between the typical social group size of a species and the size of its brain – or, more strictly, the size of its neocortex. The neocortex (literally, ‘new cortex') is the part of the brain that supports all the clever thinking that we do. It has evolved out of all proportion to the size of the rest of the brain in the primate lineage. In mammals as a whole, it occupies 10–40 percent of brain volume, but in primates, it begins at 50 percent and rises to 80 percent in humans. This relationship between neocortex size and group size in primates allows us to estimate the equivalent “natural” group size for humans: it is simply a matter of plugging human neocortex size into the equation for the monkeys and apes, and then reading off the corresponding group size. The group size for humans predicted by this equation is 150, to the nearest round number… The key point for us, perhaps, is that this is the typical size of hunter-gatherer communities, the form of society in which we have spent more than 95 percent of our existence as a species” (Dunbar, 2022, pp. 80–81).

Other group sizes of strikingly exact multiples of these basic numbers have been discovered (Dunbar, 2022, p. 87), and Dunbar has suggested that religions, and more specifically Doctrinal Religions, play a major role in maintaining cohesion in the current very large populations.

“At some point, there was a transition to a more formal kind of religion marked by regular places of worship, gods (who sometimes actively intervene in human affairs), religious specialists or priests (who intervene between the community and the gods, in some cases via trance-based rituals), more formal theologies, and moral codes that have divine origins – Moses receiving the tablets with the Ten Commandments directly from God on Mount Sinai, the Prophet Muhammed receiving the dictation of the Koran from God, Joseph Smith receiving the golden plates of the Book of Mormon. Most of these doctrinal religions also have origin stories, often associated with the revelatory experiences of a specific individual as founder – Zoroaster in the case of the Zoroastrians of ancient Persia; Siddhārtha Gautama for the Buddhists; Jesus Christ for Christians; the Prophet Muhammed for Islam; Guru Nanak for Sikhism. Because these religions typically have quite explicit theological doctrines, they are often known as doctrinal religions. They are also known as world religions because most of them now have very large followings spread over most of the planet (notwithstanding the fact that this is actually a very recent phenomenon)” (Dunbar, 2022, pp. 8–9).

Dunbar ends his book about religion, where the line between religion and ideology is not sharp, with the following conclusion:

“In short, it is difficult to see any convincing evidence for anything that will replace religion in human affairs. Religion is a deeply human trait. The content of religion will surely change over the longer term, but, for better or for worse, it is likely to remain with us” (Dunbar, 2022, p. 268)

It should be noted that China, which has the largest population, does not have any important superstition-based religion, and the meaning of the word “religion” surely should not be stretched to consider Mao's Little Red Book or Karl Marx's Das Kapital as holy scriptures.

In human life, for example, greed, violence, deception, superstition, and religions, based on it, may be hard to erase from human existence, but much is done to reduce these tendencies. The irrationality and counterfactual content often found in holy scriptures seem risky for humanity on the brink of a global catastrophe, or, in the recent words of the UN general secretary, a “collective suicide” (The Guardian, 18 July 2022).

### 4.3. Yuval Noah Hariri, imagined order, and scripts

In his multimillion-copy bestseller Sapiens, Harari wrote the following:

“UNDERSTANDING HUMAN HISTORY in the millennia following the Agricultural Revolution boils down to a single question: how did humans organize themselves in mass-cooperation networks when they lacked the biological instincts necessary to sustain such networks? The short answer is that humans created imagined orders and devised scripts. These two inventions filled the gaps left by our biological inheritance” Harari (2014, p. 168).

### 4.4. Doctrinal religions and scripted order

It seems obvious that spreading complex standardized stories and scripts, for example, the Bible and the Quran, among millions of individuals, was impossible before the advent of extra-individual memory (texts) and efficient copying and distribution technology. Such T-strings, i.e., sacred/holy scripture or books (each a T-string), when added to the texts (textome) of a society and thus become a part of its T-stringome, among others push for sometimes even evangelical spreading of the word (holy message) and thus for their own further copying and distribution, much like viruses in the DNA-based protein T-societies.

### 4.5. Textual viruses as T-strings to brain T-patterns

The similarity between textual T-strings and the temporal T-patterns in neuronal networks in brains may help to explain the powerful influence of textual T-strings on thinking and behavior, for example, by holy scriptures and misinformation, with their virus-like insertion into the T-stringoms of T-societies sometimes becoming a major threat (Magnusson, 2022). Misinformation during the viral epidemic COVID-19 has, thus, been considered a leading cause of death in the US.^8^

Billions of humans are greatly influenced by external T-string memory created during times of now unimaginable ignorance and brutality, but T-string memory is normally stored in countless copies of stable inert materials, so like DNA it may last for millennia, and any dangerous content can possibly be revived, copied, and distributed.

### 4.6. Medical vs. social use of DNA world information

While the medical use of genetic information typically identifies aspects of DNA or genes that influence the protein structure and/or function that correlate with medical symptoms, the present behavioral and social approach searches for analogies between the nanoscale DNA–protein mass societies and those of humans and has found the second-order T-society unique to humans. While the medical focus is mostly on intracellular polymer structures and functions, the focus here concerns analogous structures and functions (behavior) in human societies.

### 4.7. Proteomics as a source of ideas

Human modern T-societies ultimately descend from those of proteins, so proteomics may be a valuable source of ideas and insights into T-societies generally. Human T-societies have horizontal T-string transfer analogous to DNA transfer between cells. Questions also come up about money as an extremely powerful external memory, functioning somewhat like ATP in cells as little happens without it, and even about democracy in T-societies. Could the recently accessible nanoworld thus contain new keys to understanding the human exception and should modern humans, with their unique second-order T-societies, be considered almost like a new species, much as social insects vs. solitary insects?

### 4.8. Vaclav Smil and the exponential growth of knowledge

Similar to Dunbar, Harari, and Galor, that is, without any consideration of similarities or self-similarities between nano- and human-scale phenomena, Smil (2022, pp. 1–2) wrote regarding the current situation of accumulated knowledge and individual specialization:

“By the middle of the Eighteenth century two French savants, Denis Diderot and Jean le Rond d'Alembert could still gather a group of knowledgeable contributors, to sum up, the era's understanding in fairly exhaustive entries in their multi-volume Encyclopédie, ou Dictionnaire raisonné des sciences, des arts et des métiers.)… In 1872, a century after the appearance of the last volume of the Encyclopédie, any collection of knowledge had to resort to the superficial treatment of a rapidly expanding range of topics, and, one and a half centuries later, it is impossible, to sum up, our understanding even within narrowly circumscribed specialties: such terms as “physics” or “biology” are fairly meaningless labels, and experts in particle physics would find it very hard to understand even the first page of a new research paper in viral immunology. Obviously, this atomization of knowledge has not made any public decision-making easier. Highly specialized branches of modern science have become so arcane that many people employed in them are forced to train until their early or mid-thirties in order to join the new priesthood.”

How easy will it be for such individuals to collaborate on current global issues? Therefore, analogous to the successful nanoscale transition from the RNA to the DNA world, the success of human T-societies has been decisive as in the year 1900 knowledge doubled once a century, but now it appears, closer to once a day^9^ and with incomparably better public access to knowledge through new technology. But unfortunately, this may also be a cause for major human worries such as overpopulation and global warming.

The world of living creatures first switched from one polymer T-string world to another, from the all-RNA world to the current exclusively DNA world, thus separating information storage from work, then such separation happened in human mass societies, and a naked ape became a “text ape” or “string-ape” nearly everywhere enabled and controlled by texts.

The main conclusion here concerns the aim of this study. Beginning over half a century ago, there was an attempt to find something that clearly and non-trivially separates modern humans, and especially their modern mass societies, from all other animal species.

Surprises happened repeatedly on the way from the definition and detection of T-patterns and T-strings at multiple levels and scales to the definition of T-societies in proteins and humans. This allowed a biomathematical definition of human uniqueness, the human exception in life on earth: second-order T-societies. After the invention of human extra-individual unlimited and material memory (text), humans are the only species to have become conscious of vast areas of the universe from the innermost components of matter to the microscopic components of our own bodies but also of galaxies and galaxy clusters and other astronomical scale phenomena. Therefore, the species *Homo sapiens* seems to have finally earned its name.

It seems that a paradigm change in social organization occurred in a biological eyeblink in *Homo sapiens*, from Interactive Emergence Societies (IES), common to all non-human animals and illiterate humans, to second-order T-societies, i.e., T-societies of T-societies, never before appearing on Earth.

Where do T-patterns come from? Which came first; temporal or spatial? Can folded proteins and atoms with their electron orbits be seen as recurrent 3-D T-patterns?

This half-a-century search may eventually be elaborated and continued toward a proper T-pattern self-similarity theory, especially of mass societies, where biology and culture are one on the same biomathematical continuum.

## Data availability statement

The original contributions presented in the study are included in the article/supplementary material, further inquiries can be directed to the corresponding author.

## Author contributions

The author confirms being the sole contributor of this work and has approved it for publication.

## Footnotes

^1^ https://www.nature.com/scitable/definition/ribosome-194/

^2^See https://www.nobelprize.org/prizes/chemistry/2009/press-release/

^3^See https://www.nobelprize.org/prizes/chemistry/2009/illustrated-information/

^4^ https://www.ncbi.nlm.nih.gov/books/NBK26876/

^5^The use of the word “giant” is borrowed from the title of Grossberg and Alexei (1997).

^6^See https://www.genome.gov/genetics-glossary/Non-Coding-DNA

^7^See https://www.weforum.org/agenda/2022/09/reading-writing-global-literacy-rate-changed/.

^8^See https://www.cnn.com/videos/health/2022/05/07/fda-robert-califf-intv-misinformation-death-sot-vpx.cnn.

^9^See https://www.linkedin.com/pulse/human-knowledge-doubling-every-12-hours-amitabh-ray/.
